# IL-6-C/EBPβ signaling drives monocytic differentiation of murine cultured lymphoid progenitors with immunoregulatory properties

**DOI:** 10.1038/s41419-025-07930-4

**Published:** 2025-08-12

**Authors:** Yohei Kawano, Nozomi Katsuya, Mizuki Moriyama, Shun Ohki, Yasuo Kitajima, Tomoharu Yasuda

**Affiliations:** Department of Immunology, Graduate School of Biomedical and Health Sciences, Hiroshima University, 1-2-3 Kasumi, Minami-Ku, Hiroshima, 734-8551 Japan

**Keywords:** Myelopoiesis, Differentiation

## Abstract

While lymphoid progenitors have demonstrated unexpected plasticity in vivo, their differentiation into myeloid cells under in vitro conditions has been largely dismissed as an artifact or biologically irrelevant. Consequently, the functional properties of these cells remain poorly characterized. In this study, we show that cultured common lymphoid progenitors (cCLPs) differentiate into CD11b⁺CD115⁺ monocytic cells (cCLP-Ms) via IL-6-C/EBPβ signaling. Molecular and phenotypic analyses revealed that cCLP-Ms acquire essential features of myeloid cells, including innate immune sensor expression and phagocytic capacity, while retaining unique characteristics distinct from bone marrow-derived macrophages (BMDMs), such as reduced MHC class II expression and TNF-α production. Functionally, cCLP-Ms exhibit immunoregulatory properties, effectively suppressing IgE-mediated cutaneous allergic inflammation upon adoptive transfer. These findings highlight the plasticity of lymphoid progenitors and establish a robust platform for investigating the mechanisms underlying myeloid differentiation. This system deepens our understanding of hematopoietic cell lineage flexibility and offers a foundation for exploring therapeutic applications in immune regulation and inflammation.

## Introduction

Hematopoiesis has traditionally been conceptualized as a hierarchical process where hematopoietic stem cells (HSCs) differentiate into distinct blood cell lineages through progressively restricted progenitor states. In this classical model, HSCs give rise to multipotent progenitors (MPPs), which further differentiate into myeloid lineages including common myeloid progenitors (CMPs), and lymphoid lineages including common lymphoid progenitors (CLPs) through lymphoid-primed multipotent progenitors (LMPPs) [1, 2]. However, recent advances in single-cell analysis technologies have fundamentally challenged this paradigm, revealing that early hematopoiesis is better described as a continuous process where stem and progenitor cells gradually acquire lineage-specific characteristics [2, 3]. This revised understanding emphasizes the heterogeneity and plasticity inherent in hematopoietic differentiation, suggesting that lineage commitment is more flexible and complex than previously appreciated.

Monocytes and macrophages are essential components of the innate immune system, serving as critical sentinels in host defense against pathogens [4]. These cells express various pattern recognition receptors (PRRs), including Toll-like receptors (TLRs), NOD-like receptors (NLRs), RIG-like helicase (RLHs), and components of the inflammasome, which enable them to recognize pathogen-associated molecular patterns (PAMPs) and danger-associated molecular patterns (DAMPs) [5]. Upon activation of these innate sensors, monocytes and macrophages initiate inflammatory responses through the production of cytokines and chemokines [4, 5]. Conventionally, monocytes and macrophages arise from myeloid progenitors through a well-defined pathway involving CMPs, granulocyte-macrophage progenitors (GMPs), and monocyte-dendritic cell progenitors (MDPs). The differentiation and function of these cells are regulated by key transcription factors such as PU.1, C/EBPα, and IRF8, while cytokines including M-CSF, GM-CSF, and IL-34 control their survival and functional maturation [1, 4]. BMDMs, generated by culturing bone marrow cells with M-CSF, have served as a valuable model system for studying macrophage biology and function in numerous research applications [6–8].

Accumulating evidence has revealed unexpected plasticity in lymphoid progenitor populations, challenging the traditional view of strict lineage restriction. While LMPPs are known to retain myeloid potential [9], the extent of this plasticity in more committed lymphoid progenitors has been a subject of intensive investigation. CLPs, initially identified by Lineage-marker-negative (Lin^-^) cKit^lo^Sca-1^lo^IL-7Rα^hi^ and subsequently refined as Lin^-^cKit^int^Sca-1^int^IL-7Rα^+^Flk2^+^(CLP^f^), lack any myeloid potential and equally give rise to all lymphocytes including T, B, and natural killer (NK) cells [10, 11]. Notably, within re-defined Lin^-^Flk2^+^IL-7Rα^+^ CLP subsets, CD27^+^Ly6d^-^ all-lymphoid progenitors (ALPs) retain low levels of myeloid differentiation potential under physiological conditions, whereas more committed CD27^+^Ly6d^+^ B-cell-biased lymphoid progenitors (BLPs) lack this capability [12]. Furthermore, even committed lymphoid progenitors have shown the ability to adopt myeloid fates in vivo as well as in vitro [13–16], although the functional properties and physiological relevance of such cells remain largely unexplored.

To facilitate detailed investigation of lymphoid progenitor plasticity, we previously established a novel primary culture system enabling the long-term expansion of murine Lin^-^Flk2^+^IL-7Rα^+^CD27^+^Ly6d^-^ uncommitted CLPs under defined serum-free conditions supplemented with specific cytokines, including Flt3 ligand (Flt3L) and IL-7, without any genetic manipulation. This system not only maintains the lymphoid differentiation potential of these cultured CLPs (cCLPs) but also provides several unique advantages for studying cellular plasticity and differentiation mechanisms. The use of cCLPs enables precise tracking of differentiation processes and allows for detailed molecular mechanistic studies that would be technically challenging with CLPs in vivo or ex vivo due to their rarity.

Our previous studies revealed that these cCLPs can differentiate into myeloid cells with phagocytic activities in vitro. The present study aims to comprehensively characterize these cCLP-derived myeloid cells, focusing on their phenotypic features, molecular regulation for their differentiation, and functional properties.

## Results

### IL-6 is critical for the generation of CD11b^+^CD115^+^ monocytic cells from cCLPs

**Fig. 1:**
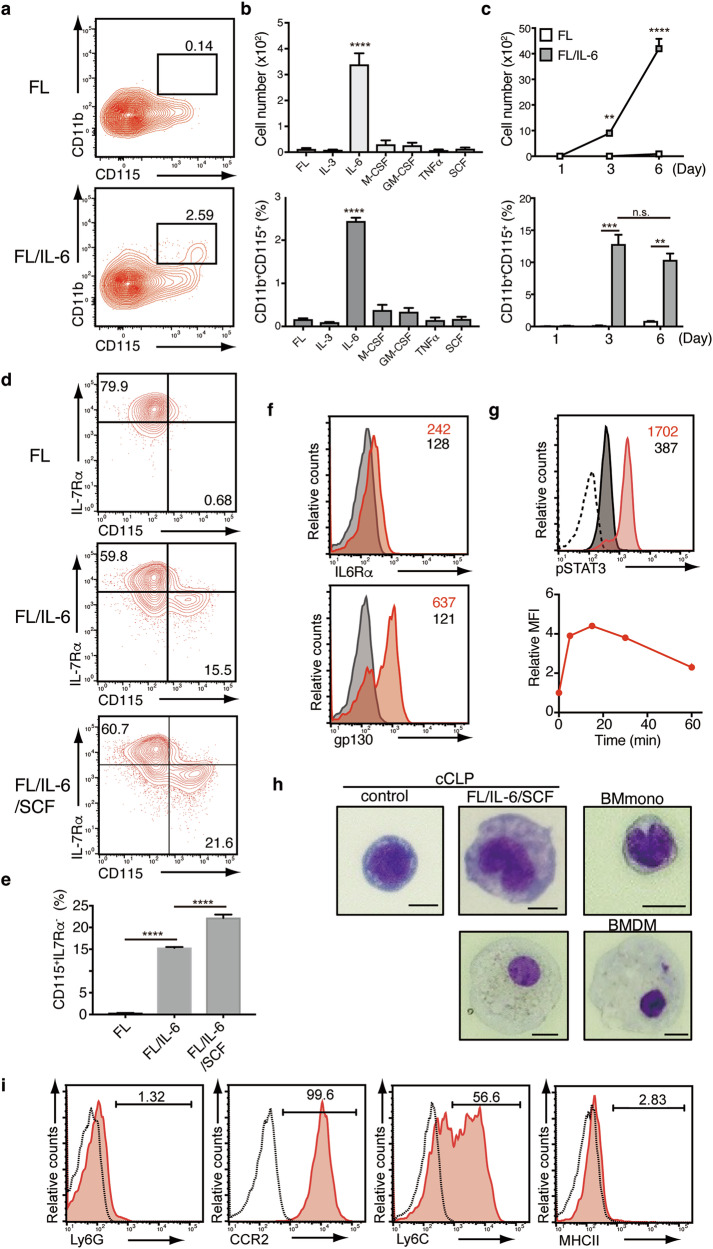
IL-6 rather than M-CSF efficiently induces a CD11b^+^CD115^+^ myeloid lineage from cCLPs. **a**–**c** cCLPs generated from Lin^-^Flk2^+^IL7Rα^+^CD27^+^Ly6d^-^ cells in murine bone marrow were maintained in cCLP medium for 3 weeks prior to the following experiments. **a** Representative FACS profiles of CD11b and CD115 expression 3 days after culturing 3000 cCLPs with Flt3L (FL) alone (*upper panel*) or together with IL-6 (FL/IL-6) (*lower panel*). The percentage in the gate is shown. **b**, **c** Cell number (*upper panel*) and percentage (*lower panel*) of CD11b^+^CD115^+^ cells on day 3 after culture with the indicated cytokines (*n* = 3 each) or at the indicated time points (*n* = 4 each) with FL (*open squares*) or FL/IL-6 (*closed squares*). **d** Representative FACS profiles on IL-7Rα and CD115 expression on day 7 in cCLPs cultured with the indicated cytokines. The percentages in the gate are shown. **e** Percentage of CD115^+^IL-7Rα^-^ cells (*n* = 3 each) in (**d**). **f** Histograms of IL-6Rα (*upper panel*) and gp130 (*lower panel*) expression (*red histograms*) on cCLPs, with isotype control shown in *gray*. Mean fluorescence intensity (MFI) values are shown in each panel. **g** pSTAT3 expression in cCLPs at 0 (*gray*) or 15 min (*red*) after the addition of IL-6 (100 ng/ml). The dotted line represents the isotype control. **h** May-Grünwald Giemsa staining images of cCLPs (control, *left panel*) or CD11b^+^CD115^+^Ly6C^+^CCR2^+^ cells isolated by FACS 4 days after cCLP culture with FL/IL-6/SCF (*middle panels*). The right panels show a representative image of CD11b^+^CD115^+^Ly6G^-^Ly6C^+^CCR2^+^ monocytes isolated ex vivo (BMmono) or cultured macrophages (BMDMs) from the bone marrow of C57BL/6 mice. Scale bar = 10 μm. **i** cCLPs were cultured with 10 ng/mL of FL/IL-6/SCF for 4 days. A representative FACS profile of indicated molecules. The percentages in the gate and histograms are shown. Data are mean ± SD with statistical significance determined by one-way ANOVA (in **b**, **e**) or two-way ANOVA (in **c**). The p-values are represented as **, <0.01; ***, <0.001; ****, <0.0001. n.s., not significant. All data are representative of at least two independent experiments.

We previously demonstrated that cCLPs can induce the development of CD11b^+^ myeloid cells with phagocytotic function when exposed to a complex cytokine mixture [17]. To delineate the key factors responsible for driving myeloid lineage differentiation, we evaluated individual cytokines and their combinations using 3 weeks-cultured cCLPs freshly generated from Lin^-^Flk2^+^IL7Rα^+^CD27^+^Ly6d^-^ bone marrow cells. Strikingly, IL-6 emerged as the primary driver of CD11b⁺CD115⁺ cell development, surpassing the effects of canonical myeloid factors such as M-CSF (Fig. 1a, b). In the presence of Flt3L (FL), which is essential for cCLP survival [17], IL-6 induced the generation of CD11b⁺CD115⁺ cells, reaching approximately 12% of the population by day 3. This population expanded further, achieving numbers over the initial cCLP input by day 6 (Fig. 1c). Consistent findings were also observed in cryopreserved cCLPs maintained for 2 months in culture (Supplementary Fig. S1a). These cCLPs retained the differentiation potential to lymphoid cells such as B cells as previously reported (Supplementary Fig. S1b) [17]. The differentiation of these CD11b⁺ myeloid cells was substantially affected by the type of FBS used, whereas B cell differentiation was less influenced by FBS (Supplementary Fig. S1c). The myeloid differentiation process was characterized by reciprocal regulation of key lineage markers: downregulation of IL-7Rα, essential for lymphoid identity, concurrent with upregulation of CD115, a defining marker of the monocyte/macrophage lineage. The addition of SCF enhanced this phenotypic conversion (Fig. 1d, e). Furthermore, supplementation with all-trans retinoic acid (ATRA) augmented both the frequency and absolute numbers of CD11b⁺CD115⁺ cells, suggesting a synergistic effect in promoting myeloid differentiation (Supplementary Fig. S1d, e). Mechanistically, we found that cCLPs express both components of the IL-6 receptor complex—IL-6Rα and gp130—enabling direct response to IL-6 stimulation. Time-course analysis revealed rapid STAT3 phosphorylation following IL-6 exposure, indicating active signal transduction through this pathway (Fig. 1f, g). These results suggest that IL-6 signaling initiates the myeloid differentiation program by modulating the balance between lymphoid maintenance (IL-7Rα) and myeloid specification (CD115) pathways.

The resulting CD11b⁺CD115⁺ cells exhibited classical monocyte/macrophage characteristics. Morphological analysis using May-Grünwald-Giemsa staining revealed cells with kidney-shaped nuclei and abundant cytoplasm, hallmark features of monocytes (Fig. 1h, *upper panels*). A subset of cells displayed even more extensive cytoplasmic development, resembling BMDMs (Fig. 1h, *lower panels*). Flow cytometric analysis confirmed a surface marker profile typical of monocytes: Ly6C⁺^/^⁻Ly6G⁻CCR2⁺MHC class II⁻ (Fig. 1i). Furthermore, the in vivo transfer experiment showed that cCLPs gave rise to CD11b⁺CD115⁺ cells in LPS-treated mice, but not in PBS-treated mice, ruling out that the myeloid differentiation potential of cCLPs is due to specialized culture conditions. (Supplementary Fig. S1f). Collectively, these findings demonstrate that IL-6 signaling can direct lymphoid progenitors toward a monocytic fate, generating cells that closely resemble conventional monocytes/macrophages in both phenotype and morphology.

### Lymphoid progenitors show myeloid potential ex vivo under specific cytokine milieu

**Fig. 2:**
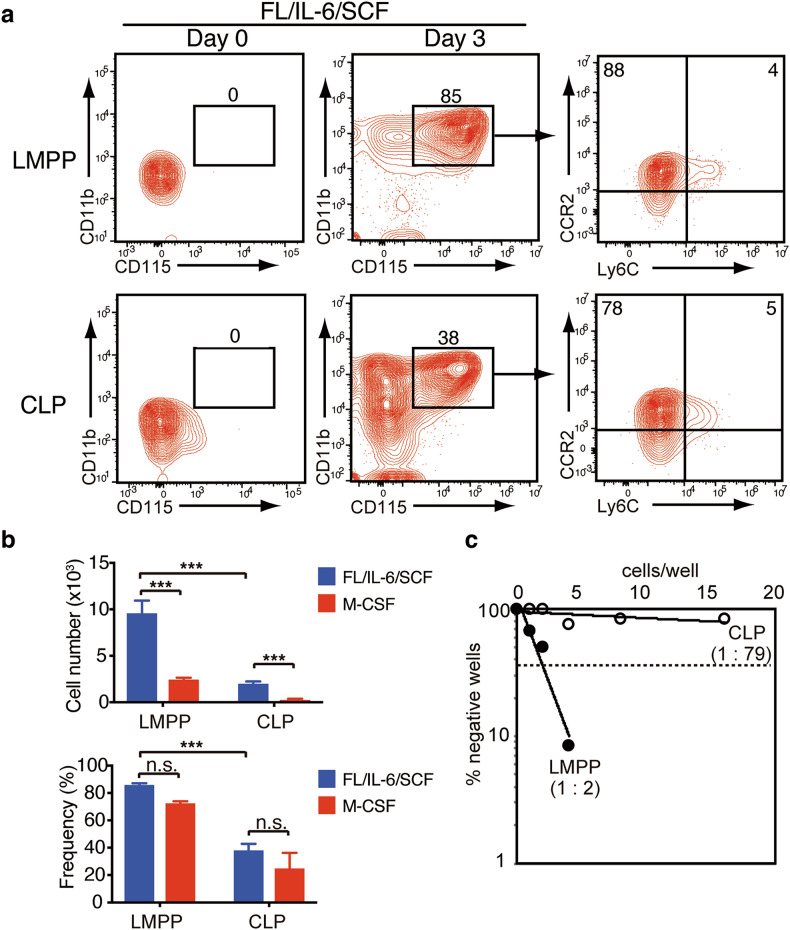
Lymphoid progenitors from murine bone marrow can differentiate to myeloid lineages upon culture with FL/IL-6/SCF. LMPPs and uncommitted CLPs were isolated from the bone marrow of C57BL/6 mice and cultured with 10 ng/mL of FL/IL-6/SCF or 10 ng/ml of M-CSF for 3 days. **a** A representative FACS profile of indicated molecules before *(left panels*) and after *(middle and right panels*) culture of 1000 LMPPs (*upper panels*) or CLPs (*lower panels*) with FL/IL-6/SCF. The percentages in the gate are shown. **b** The number (*upper panel*) and frequency (*lower panel*) of CD11b^+^CD115^+^ cells after culture (*n* = 3, each). **c** Limiting dilution assay. LMPPs (*closed symbols*) and CLPs (*open symbols*) serially diluted from 16 cells to 1 cell by 2-fold are shown. Data are mean ± SD with statistical significance determined by two-way ANOVA with multiple comparisons. The *p* values are represented as ***, <0.001. n.s. not significant. All data are representative of at least two independent experiments.

To determine whether the observed lineage plasticity represents a general property of lymphoid progenitors rather than a culture-induced phenomenon, we examined the myeloid potential of freshly isolated progenitor populations, uncommitted CLPs, and LMPPs from mouse bone marrow. When cultured with FL/IL-6/SCF, both primary populations generated CD11b⁺CD115⁺ cells within 4 days. These cells exhibited the characteristic CCR2⁺Ly6C⁺^/^⁻ monocytic phenotype, mirroring our observations with cCLPs (Fig. 2a). Importantly, both progenitors generated greater number of CD11b^+^CD115^+^ cells under FL/IL-6/SCF conditions rather than with M-CSF alone (Fig. 2b). Limiting dilution analysis further demonstrated that LMPPs had a higher clonal potential for differentiation into CD11b^+^CD115^+^ cells than CLPs (Fig. 2c). Overall, these results suggest that primary lymphoid progenitors possess intrinsic myeloid potential that can be accessed under specific cytokine conditions.

### Comprehensive gene expression profiling characterizes that cCLP-derived monocytic cells are closely related to monocytes/macrophages

**Fig. 3:**
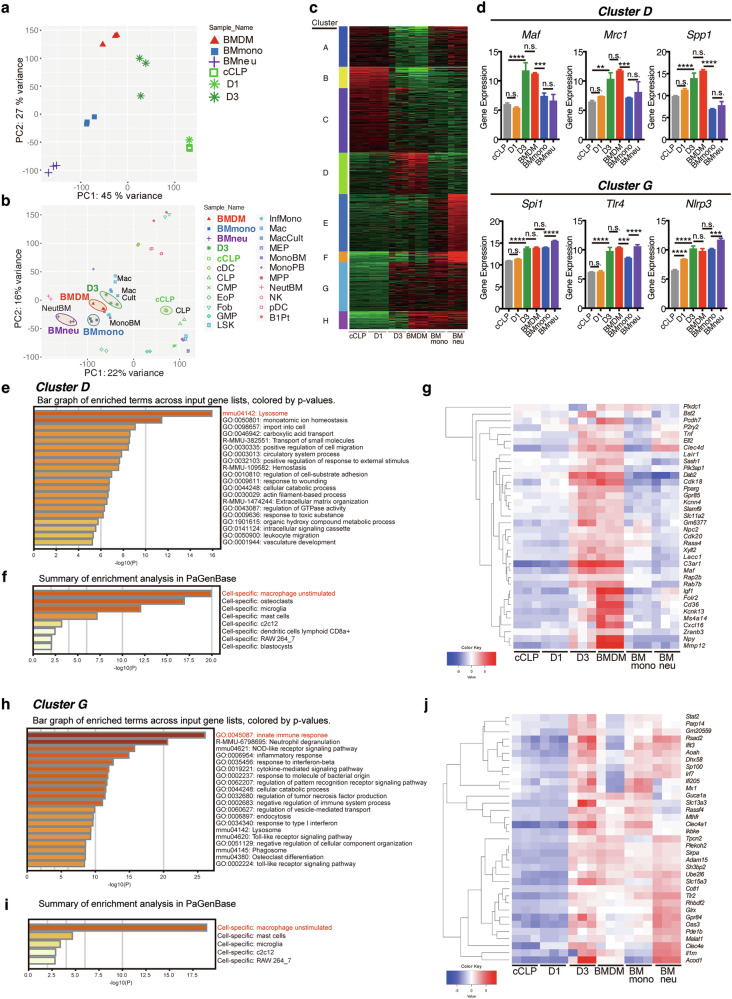
cCLP-derived myeloid cells display gene expression signatures resembling monocytes/macrophages. **a** PCA of whole transcripts of 15,074 genes with CPM values greater than 1 in either sample across all samples from cCLPs, and those culture with FL/IL-6/SCF for 1 day (D1) or 3 days before the isolation of CD11b^+^CD115^+^CCR2^+^Ly6C^+^Ly6G^-^ (D3), together with BMDMs, BMmono, and BMneu. *n* = 3, each. **b** PCA plots of whole transcripts in indicated cell populations merged with a publicly available database of murine hematopoietic cells (GSE116177). **c** k-means clustering analysis of 6500 genes with SD > 1 across all samples. The color bar on the left shows each cluster; A (*n* = 773), B (*n* = 502), C (*n* = 1387), D (*n* = 936), E (*n* = 1255), F (*n* = 259), G (*n* = 998), H (*n* = 390). **d** The expression levels of the indicated genes derived from RNA-seq data representative in Cluster D (*upper panels*) and Cluster G (*lower panels*). The values are shown as log_2_ (CPM + 4). Data are mean ± SD with statistical significance determined by one-way ANOVA with multiple comparisons. The *p* values are represented as *, <0.05; **, <0.01; ***, <0.001; ****, <0.0001. n.s., not significant. Enrichment analysis of terms (**e**, **h**) and cell population (**f**, **i**) by Metascape and the heatmap analysis of gene express**i**on related to macrophages (**g**, **j**) enriched in (**f**) or (**i**) of Cluster D or G, respectively. Color code values indicate the Z-score for each gene across samples showing the log_2_ fold change for each gene.

To assess the transcriptional signatures of cCLP-derived myeloid cells, RNA-seq-based whole transcriptome analysis was performed to compare the gene expression profile with that of BMDMs from cell culture as well as of CD11b^+^Ly6G^+^ neutrophils (BMneu) or CD11b^+^Ly6C^hi^ monocytes (BMmono) isolated ex vivo from bone marrow (Supplementary Fig. 2a–d). In a principal component analysis (PCA) of the whole transcriptome, comprising 15,074 expressed genes and accounting for approximately 70% of the variance across the PC1 and PC2 axes, the cell population derived from cCLPs a day after the induction of differentiation with FL/IL-6/SCF (D1) was located almost in the same position as cCLPs. In contrast, the population 3 days after the induction of differentiation (D3) was positioned closest to BMDMs and nearer to BMmono than to BMneu. These results suggest that cCLPs cultured in the presence of FL/IL-6/SCF acquire a myeloid gene expression profile closely aligned with the monocyte lineage (Fig. 3a). To place these findings in a broader hematopoietic context, we integrated our data with a comprehensive mouse hematopoiesis database (GSE116177) [18]. This expanded analysis confirmed our previous observation that unstimulated cCLPs closely resembled fresh CLPs [17]. More importantly, it revealed that D3 cells clustered with cultured macrophages and bone marrow monocytes from both our preparation and the reference dataset, further validating their monocytic identity (Fig. 3b).

Unsupervised k-means clustering identified eight distinct gene expression patterns including particularly informative clusters associated with the differentiation process (Fig. 3c). Cluster D (936 genes) captured a macrophage-specific signature shared between D3 cells and BMDMs. This included *Spp1*, encoding a secreted factor characteristic of macrophages [19], and several genes associated with immunosuppressive, M2-like macrophages, such as the transcription factor *Maf* (also known as c-Maf) [20] and the surface marker *Mrc1* (Fig.3d). Cluster G (998 genes) represented a broader myeloid program shared among D3 cells, BMDMs, BMmono, and BMneu (Fig. 3c). This cluster encompassed key regulators of both lymphoid and myeloid differentiation, including the master transcription factor PU.1 (*Spi1*). It also contained numerous components of innate immune sensing pathways: inflammasome proteins (*Nlrp3*, *Nlrc4*), intracellular pattern recognition receptors (*Nod2*, *Ddx58*), and multiple Toll-like receptors (*Tlr1*, *Tlr2*, *Tlr4*, *Tlr6*, and *Tlr13*) (Fig. 3d and Supplementary Fig. 3). Further enrichment analysis revealed that genes related to ‘Lysosome’ and ‘Ion homeostasis’ were enriched in Cluster D (Fig. 3e), with PaGenBase enrichment analysis identifying 36 genes as macrophage-enriched, including *P2ry2* and *Pparg* (Fig. 3f, g). Similarly, Cluster G genes were associated with ‘Innate immune response’ (Fig. 3h) and included 35 macrophage-enriched genes such as *Irf7* and *Mx1*, critical for antiviral responses (Fig. 3i, j). Importantly, the majority of macrophage-related genes enriched in Cluster D and Cluster G were expressed at low levels in cCLPs and D1 cells (Fig. 3g, j). Collectively, these results suggest that cCLP-derived myeloid cells induced by FL/IL-6/SCF over three days acquire a gene expression program characteristic of innate immune responses, including pathogen recognition sensors and lysosomal molecules involved in intracellular digestion. This gene expression signature, resembling that of monocytes/macrophages, indicates differentiation from the lymphoid to myeloid lineage at the transcriptional level. Therefore, we refer to these cells as cCLP-Ms (cCLP-derived monocytic cells).

### Investigation of transcriptional regulators critical for cCLP-M induction identifies C/EBPβ

**Fig. 4:**
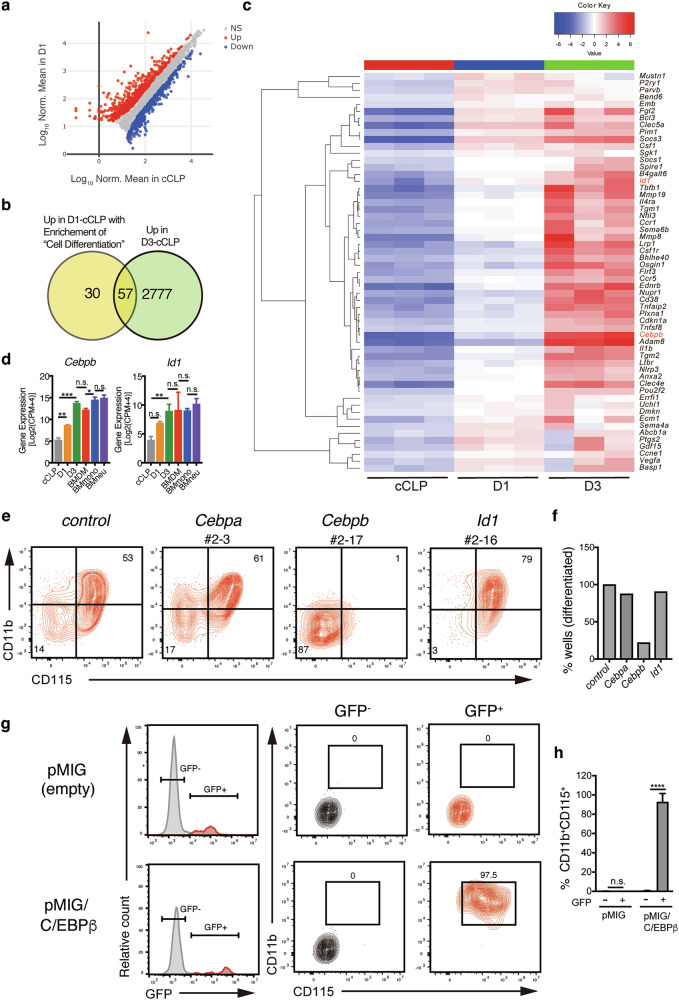
Investigation of transcriptional regulators critical for cCLP-M induction identifies C/EBPβ. **a** Scatter plot of DEG (FDR < 0.05, >2-fold or <0.5- fold) between undifferentiated cCLPs and cCLPs after 24 h incubation with FL/SCF/IL-6 (D1). **b** Venn diagram of genes up-regulated in D1 to cCLPs with enrichment analysis of “Cell Differentiation” by GO Biological process and those up-regulated in D3 to cCLPs. **c** Heatmap view of 57 genes overlapped in (**b**). Color code values indicate the Z-score for each gene across samples. **d** Gene expression of *Cebpb* (*left panel*) and *Id1* (*right panel*) in indicated samples. The values are shown as log_2_ (CPM + 4) from RNA-seq data. **e** Representative FACS plots of cCLP-Ms differentiated from single cCLP clones where indicated genes were targeted by Crispr/Cas9. The name of a representative clone targeted to each gene is shown (＃2–3, ＃2-17, #2-16). **f** Frequency of wells with more than 100 live cells in (**e**). **g** Frequency of wells with less than 40% CD11b^+^CD115^+^ cells. **h** Representative histogram of GFP expression (*left panels)*, plots for CD11b and CD115 expression on GFP^-^ gate (*middle panels*), or GFP^+^ gate (*right panels*) in cCLPs on day 4 after retroviral transduction with pMIG empty vector (*upper panels*) or pMIG/ C/EBPβ (*lower panels*). **i** Percentages of CD11b^+^CD115^+^ cells in the indicated fractions in (**h**). Data are mean ± SD with statistical significance determined by one-way ANOVA (in **d**) or two-way ANOVA (in **i**) with multiple comparisons. The *p* values are represented as **, <0.01; ***, <0.001; ****, <0.0001. n.s. not significant.

To identify the transcriptional regulators driving cCLP-M differentiation, we performed differential expression analysis comparing cCLPs with cells after one day of IL-6 stimulation (D1). This analysis revealed 216 upregulated and 177 downregulated genes (fold change >2 or <0.5, *p* < 0.05) in D1 cells (Fig. 4a). GO pathway enrichment analysis indicated that 87 of the upregulated genes were associated with the GO biological process “Cell differentiation” (Supplementary Tables 1, 2). Remarkably, 57 of these genes maintained elevated expression in fully differentiated cCLP-Ms (D3), representing a core set of differentiation-associated factors (Fig. 4b). Among these 57 candidate genes, we focused on the transcription factors Id1 and C/EBPβ (Fig. 4c), both of which have been previously implicated in IL-6-mediated responses [21]. The expression levels of these genes were upregulated in D1 (*Id1*: ~8-fold, *Cebpb*: ~8-fold) and further increased in cCLP-Ms (D3) (*Id1*: ~32-fold, *Cebpb*: ~400-fold), reaching levels comparable to those observed in BMDMs, BMmono, and BMneu (Fig. 4d). To test the functional requirement for these factors, we employed CRISPR-Cas9-mediated gene editing to target *Id1*, *Cebpb*, or its relative *Cebpa* in cCLPs. Following clonal expansion (Supplementary Fig. 4a), we observed distinct effects on cell proliferation and survival. While control (mock) and *Id1*-targeted clones showed robust expansion with all clones exceeding 100 cells, *Cebpa*-targeted clones showed reduced viability, with only 89% (16/18 clones) achieving similar expansion. *Cebpb*-targeted clones were even more severely affected, with only 50% (9/18 clones) showing substantial growth (Supplementary Fig. 4b).

The impact on differentiation capacity was even more striking. When induced toward monocytic differentiation, control clones consistently generated substantial CD11b⁺CD115⁺ populations ( > 40% in all 18 clones). Similarly, most *Id1*-targeted (14/16 clones) and *Cebpa*-targeted (19/21 clones) clones retained differentiation capacity. In contrast, only 22% (2/9 clones) of viable *Cebpb*-targeted clones produced significant CD11b⁺CD115⁺ populations (Fig. 4e, f), suggesting the critical contribution of C/EBPβ in both the differentiation and the survival for cCLP-Ms. To determine whether C/EBPβ was sufficient to drive monocytic differentiation, we performed gain-of-function experiments using retroviral expression. Strikingly, enforced expression of *Cebpb* in cCLPs induced CD11b⁺CD115⁺ populations even in the absence of IL-6 or other inducing cytokines (Fig.4g, h). Taken together, these results indicate that C/EBPβ is a key regulator sufficient for cCLPs to differentiate into cCLP-Ms, implicating it as a pivotal factor in regulating lineage commitment within this system.

### cCLP-Ms exhibit distinct functional properties and immunoregulatory capacity

**Fig. 5:**
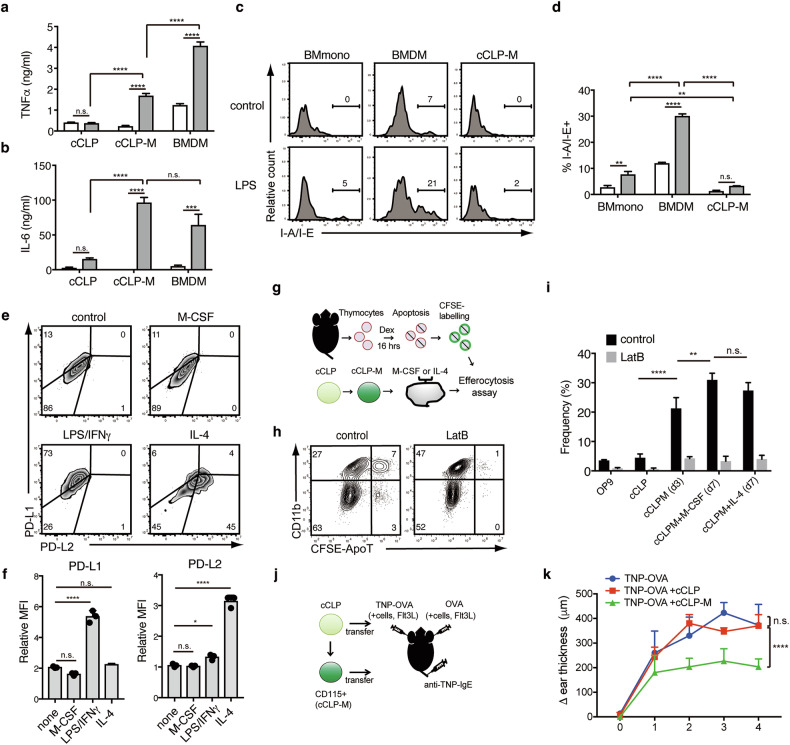
cCLP-Ms function in vitro and in vivo*.* **a**–**d** The indicated cells were stimulated with (*filled bars*) or without (*open bars*) LPS (1 μg/mL) for 24 h. Culture supernatants and cells were harvested for ELISA and FACS, respectively. TNFα and IL-6 production, representative histograms for MHC class II (I-A/I-E) expression, and the percentage of I-A/I-E^+^ cells are shown (*n* = 3, each). **e** Representative FACS plots and **f** the relative MFI of PD-L1 and PD-L2 expression on cCLP-M three days after culture with M-CSF, LPS /IFN-γ, IL-4, or medium alone (control) (*n* = 3, each). **g** Experimental scheme for efferocytosis assay. Dex: Dexamethasone. **h** Representative FACS plots for CFSE and CD11b expression on cCLP-Ms. **i** The percentage of CFSE-positive cells among indicated populations, or within the CD11b^+^ subset for cCLP-Ms and those cultured with M-CSF or IL-4 (*n* = 4, each). Among each population, the groups treated with DMSO (control) or Latrunculin B (LatB) are shown in *dark gray* and *light gray bars*. **j** Experimental scheme for IgE-CAI in mice transferred with cCLP-Ms (**k**). Time course of ear swelling (Δ ear thickness) after the challenge with TNP-OVA (antigen) alone (in *blue*) or combined with the transfer of cCLPs (in *red*) or cCLP-Ms (in *green*) in anti-TNP IgE-sensitized mice (*n* = 3 each). Data are mean ± SD with statistical significance determined by one-way ANOVA (in **f**) or two-way ANOVA with multiple comparisons (in **a**, **b**, **d**, **i**) or tukey’s multiple comparisons test (in **k**). The *p* values are represented as *, <0.05; **, <0.01; ***, <0.001; ****, <0.0001. n.s., not significant. All data are representative of at least two independent experiments.

Having established the molecular basis of cCLP-M generation, we next investigated their functional capabilities. As our transcriptional analysis had revealed robust expression of innate immune sensors in cCLP-Ms, including *Tlr4* (Fig. 3c), we first examined their response to TLR ligands. When stimulated with the TLR4 ligand LPS, cCLP-Ms produced significant amounts of IL-6 at levels comparable to BMDMs, but notably lower levels of TNF-α (Fig. 5a, b). Other TLR ligands such as R848 (TLR7) and ODN2395 (TLR9) also elicited these cytokine production from cCLP-Ms but not from cCLPs, despite comparable expression of *Tlr7* and *Tlr9* (Supplementary Fig. 5a–c). Interestingly, cCLP-Ms also produced IL-10 in response to R848 and ODN2395, but not to LPS, suggesting selective activation of anti-inflammatory pathways (Supplementary Fig. 5d). A striking feature of cCLP-Ms was their limited MHC class II expression, which remained low even after TLR stimulation, contrasting sharply with the robust upregulation observed in BMDMs and BMmono (Fig. 5c, d, and Supplementary Fig. 5e). These findings suggest that cCLP-Ms displays distinct potential on cytokine secretion and molecular expression in response to TLR ligands compared to BMDMs and BMmono.

To explore their functional plasticity, we examined whether cCLP-Ms could undergo classical M1/M2 polarization, a hallmark of macrophage versatility [22]. Whereas stimulation with LPS and IFN-γ induced the upregulation of the M1 marker PD-L1, IL-4 stimulation led to increased expression of the M2 marker PD-L2 in cCLP-Ms [23], indicating that cCLP-Ms have the plasticity to undergo M1/M2 polarization (Fig. 5e, f). We next assessed their phagocytic capability through an efferocytosis assay, measuring the uptake of apoptotic thymocytes (Fig. 5g). Unlike cCLPs or OP9, cCLP-Ms efficiently bound CFSE-labeled apoptotic cells in an actin-dependent manner, as demonstrated by inhibition with Latrunculin B (Fig. 5h, i). This phagocytic capacity was further enhanced by M-CSF or IL-4 treatment, indicating that cCLP-Ms can acquire advanced macrophage functions through additional maturation signals.

A previous report showed that inflammatory monocytes play a critical role in ameliorating IgE-dependent cutaneous allergic inflammation (IgE-CAI) in mice by converting into anti-inflammatory M2-type macrophages in the skin. This was demonstrated through the adoptive transfer of bone marrow-derived CD115^+^ monocytes from wild-type mice to the affected ear skin (Fig. 5j) [24]. We tested the in vivo function of cCLP-Ms in this mouse model of allergic inflammation. Remarkably, intradermal transfer of cCLP-Ms, but not cCLPs, reduced ear swelling by approximately 50% compared to control conditions (Fig. 5k). Tracking cell trace violet (CTV)-labeled cCLP-Ms following transfer revealed the presence of CTV^+^F4/80^+^PD-L2^+^ cells exclusively in the inflamed ear tissue (TNP-OVA + cCLP-M) induced by IgE-CAI (Supplementary Fig. 6). This observation aligns with previous reports, suggesting that the transferred cCLP-Ms function in vivo by differentiating into M2 macrophages within the inflammatory site, thereby contributing to the suppression of IgE-CAI.

## Discussion

In this study, we demonstrated that IL-6 stimulation drives the differentiation of CD11b⁺CD115⁺ monocytic cells (cCLP-Ms) from cCLPs. This differentiation was observed not only in cCLPs but also in primary lymphoid progenitors under similar in vitro conditions. Furthermore, in vivo transfer experiments revealed that cCLPs can give rise to CD11b⁺CD115⁺ cells following LPS administration, highlighting their latent myeloid potential under inflammatory conditions. These findings suggest that lymphoid progenitors exhibit lineage plasticity, shifting toward myeloid differentiation in response to inflammatory signals.

The cCLP-Ms displayed unique phenotypic and functional attributes that distinguish them from BMDMs, conventional cultured macrophages derived from bone marrow progenitors. While acquiring innate immune characteristics such as innate sensor expression and phagocytic activity, cCLP-Ms maintained lower levels of MHC class II and TNF-α expression, suggesting they may constitute a distinct myeloid population with unique immunological functions. Moreover, these cells demonstrated remarkable plasticity in M1/M2 polarization and immunoregulatory potential, as evidenced by their ability to suppress IgE-mediated cutaneous allergic inflammation upon adoptive transfer. These findings highlight the utility of cCLP-Ms as a valuable system to unravel the molecular mechanisms underlying the differentiation and functional properties of lymphoid-derived monocytes/macrophages.

At the molecular level, we identified C/EBPβ as a key transcription factor mediating the IL-6-induced differentiation of cCLP-Ms. Unlike conventional macrophage differentiation pathways regulated by key transcription factors such as PU.1, C/EBPα, and IRF8 [1, 4, 25], the cCLP-M differentiation process appears to be predominantly C/EBPβ-dependent as it was up-regulated ~400-fold in cCLP-Ms compared to cCLP. Notably, overexpression of C/EBPβ alone was sufficient to induce CD11b⁺CD115⁺ differentiation from cCLPs in the absence of IL-6. These results align with previous reports linking IL-6 to STAT3-mediated induction of C/EBPβ and its downstream regulation of *Csf1r* (CD115) transcription [26–28]. C/EBPβ is known to regulate specialized myeloid populations, such as Ly6C^lo^ non-classical monocytes and segregated-nucleus-containing atypical monocytes (SatM) [28, 29]. It also contributes to the development of resident macrophages, such as large peritoneal macrophages (LPM), which exhibit high CD11b and F4/80 expression but low MHC class II levels [30]. The involvement of C/EBPβ in cCLP-M differentiation suggests potential molecular links with these specialized populations, warranting further investigation into their transcriptional programs.

IL-6 stimulation markedly reduced IL-7Rα expression, a receptor essential for lymphoid progenitor survival and differentiation [31, 32], shedding light on the mechanisms underlying lymphoid-to-myeloid transition. IL-7Rα expression is regulated by transcription factors such as PU.1, which plays a pivotal role in both lymphoid and myeloid differentiation [33, 34]. High PU.1 levels favor myeloid differentiation, while low levels promote lymphoid development [35]. PU.1 is also required for the developmental progression of multipotent progenitors to common lymphoid progenitors [36]. Notably, PU.1 has been reported to interact with C/EBPβ to regulate monocyte/macrophage gene expression in addition to its association with C/EBPα on lineage commitment to macrophages [37–39]. Therefore, IL-6 stimulation may deplete low-level PU.1 required for IL-7Rα expression, thereby driving myeloid differentiation, or that C/EBPβ directly or indirectly suppresses IL-7Rα expression. These molecular mechanisms warrant further investigation to elucidate their contributions to lineage plasticity.

While our data present compelling evidence for IL-6–mediated lineage reprogramming, the conclusions are based primarily on in vitro culture systems. However, these findings raise the possibility that similar mechanisms could occur under pathological conditions characterized by chronic inflammation, such as infections, autoimmune diseases, or cancer. In particular, the role of IL-6 in promoting myeloid-biased differentiation at the expense of lymphoid output during inflammation has been well-documented [21, 40, 41]. Recent studies have also provided evidence that macrophage-like cells (termed B-MF) are generated from B-cell precursors, a lymphoid progenitor downstream of CLPs in vivo in various murine tumor models and samples from humans with breast and ovarian cancers [42]. Additionally, human hematopoietic stem cells have been shown to differentiate into myeloid cells through the IL-6–C/EBPβ axis, consistent with our findings [43, 44]. Understanding the precise molecular pathways and contexts in which lymphoid-to-myeloid plasticity occurs could uncover novel therapeutic targets to modulate immune responses. Further investigations are required to establish whether IL-6 and C/EBPβ mediate myeloid differentiation from lymphoid progenitors under physiological or pathological conditions in vivo.

In conclusion, our study establishes a system for investigating aspects of monocyte development and reveals unexpected properties of CLP-derived myeloid cells. This system provides valuable opportunities for studying fundamental aspects of monocyte biology and the molecular mechanisms underlying myeloid cell differentiation. Our findings also highlight the importance of cell plasticity in immune cell development. While CLPs are traditionally viewed as lymphoid-committed progenitors, their ability to generate functional myeloid cells under specific conditions suggests that lineage commitment may be more flexible than previously appreciated. Although the physiological relevance of this pathway remains to be fully clarified, our results provide new insights into the diversity and plasticity of myeloid cell development.

## Materials and methods

### Mice

All experiments utilized C57BL/6 mice obtained from CLEA Japan (Tokyo, Japan). Animals were maintained under specific pathogen-free conditions in the animal facilities of Hiroshima University and Tokyo Medical and Dental University.

### Reagents

Recombinant mouse cytokines were obtained from BioLegend (San Diego, CA, USA): Flt3L (550706), IL-7 (577802), SCF (579702), IL-3 (575504), IL-6 (575702), TNF-α (575208), M-CSF (576404), GM-CSF (576302), IL-4 (574304), and IFN-γ (575304). TLR ligands included LPS from *Escherichia coli* O111:B4 (SIGMA, St. Louis, MO, USA, L2630), R848 (Adipogen, San Diego, CA, USA, AG-CR1-3582-M005), and ODN2395 (Adipogen, IAX-200-007-C100).

### Cell lines

OP9 stromal cells were maintained in OP9 medium consisting of α-minimum essential medium (Thermo Scientific, Waltham, MA, USA, 11900-073) supplemented with 20% fetal bovine serum (FBS), 2.2 g/L NaHCO₃, and 1× penicillin-streptomycin (Nacalai Tesque, Kyoto, Japan). Cells were passaged every 3–4 days and plated at 1 × 10⁴ cells/cm² one day before use in cCLP cultures. Plat-E retrovirus packaging cells were maintained in standard Dulbecco’s Modified Eagle Medium (DMEM) culture medium [DMEM (Fujifilm, Tokyo, Japan, 043-30085), 10% FBS (Nichirei, Tokyo, Japan), 1% Penicillin/Streptomycin (Nacalai Tesque, 26253-84), and 25 mM HEPES (Fujifilm, Tokyo, Japan, H3375)] [45]. All cells were cultured in a humidified atmosphere of 5% CO_2_ at 37 °C and were confirmed to be free from mycoplasma contamination.

### Preparation and culture of cCLPs

A serum-free basal culture medium, KIDMEM, was prepared according to the previously established protocols [17]. cCLPs were generated from either freshly isolated bone marrow as previously described [17] or derived from cryopreserved stocks (1–3 ×105 cells) that had been cultured in vitro for 3-10 weeks. Cells were cultured in plates pre-coated with OP9 cells in cCLP culture medium containing KIDMEM supplemented with 25 ng/mL Flt3L and 0.1 ng/mL IL-7. Under these conditions, 70-80% of cells maintained the Lin⁻IL-7Rα⁺CD27⁺Ly6D⁻ phenotype characteristic of uncommitted progenitors. In some experiments, these undifferentiated cells were sorted for further analysis.

### Generation of bone marrow-derived macrophages (BMDMs)

BMDMs were generated by culturing bone marrow cells isolated from mouse femurs in 150-mm petri dishes (FALCON, NY, USA, 351058) in DMEM culture medium containing 20 ng/mL M-CSF. This standardized protocol consistently yields highly pure populations of functional macrophages.

### In vitro differentiation from cCLPs to CD11b^+^ myeloid cells

cCLPs were cultured for 3–4 days on OP9 cells in complete RPMI medium [RPMI1640 (Fujifilm, 189-02145), 10% FBS, 1× sodium pyruvate (Nacalai Tesque, 06977-34), 1× non-essential amino acids (Nacalai Tesque, 06344-56), 10 mM HEPES, 50 μM β-mercaptoethanol (Thermo Scientific, 21985023), and 1× penicillin-streptomycin (Nacalai Tesque, 09367-34)]. Cytokine supplementation included Flt3L (10 ng/mL), SCF (10 ng/mL), IL-3 (10 ng/mL), IL-6 (10 ng/mL), TNF-α (20 ng/mL), M-CSF (10 ng/mL), and GM-CSF (20 ng/mL) individually or in combination. To induce differentiation into cCLP-Ms, a cytokine mixture of Flt3L (10 ng/mL), SCF (10 ng/mL), and IL-6 (10 ng/mL) was applied. In some experiments, 1 μM all-trans retinoic acid (ATRA, Nacalai Tesque, 6331-44) was added to enhance differentiation efficiency. Cultures were maintained at 37 °C in a humidified atmosphere containing 5% CO₂, with regular monitoring of cell morphology and surface marker expression.

### Ex vivo culture and limiting dilution assay of lymphoid progenitor cells

Lin^-^ bone marrow cells from 7-week-old C57BL/6 mice were enriched by magnetic depletion with a lineage antibody cocktail (biotinylated CD3, CD19, B220, CD11b, CD11c, Gr-1, NK1.1, Ter-119), followed by BD IMag™ Streptavidin Particles Plus (BD Biosciences, 557812). LMPPs (Lin⁻Sca1⁺c-Kit⁺Flk2⁺IL-7Rα⁻) and uncommitted CLPs (Lin⁻Flk2⁺IL-7Rα⁺CD27⁺Ly6D⁻) were isolated using FACSAria™ II or FACSAria™ Fusion (BD Biosciences) and cultured with 10 ng/mL each of Flt3L/IL-6/SCF or 10 ng/mL M-CSF. Cells were plated on OP9-pre-coated flat-bottom plates, with 1000 cells/well in 96-well plates for 3 days, or at serial twofold dilutions (16 to 1 cell per well) for 7 days for limiting dilution assay. Cells were then harvested for flow cytometric analysis. In the limiting dilution assay, wells containing more than 10 CD11b⁺CD115⁺ cells were considered positive, and the frequency of myeloid potential for each population was determined by counting the number of negative wells and calculating frequencies based on Poisson distribution.

### Flow cytometry

Single-cell suspensions were prepared in FACS buffer PBS containing 1% FBS, 5 mM EDTA, and 0.2% sodium azide and blocked with anti-CD16/CD32 (clone 2.4G2) before staining with the following fluorochrome-conjugated anti-mouse monoclonal antibodies: FITC-labeled Ly6G (1A8, #127605), Ly6C (1G7.G10, #128006), and I-A/I-E (M5/114.15.2, #107605) from BioLegend, and PD-L1 (MIH6, MCA2626F) from AbD Serotec; PE-labeled IL-6Rα (W18166A, #160405), Flk2 (#135305), Ly6d (49-H4, #138606), I-A/I-E (M5/114.15.2, #107607), and B220 (RA3-6B2, #103207) from BioLegend; PE/Cy7-labeled CD11b (M1/70, #101215), Ly6C (1G7.G10, #128018), and IL-7Rα (A7R34, #135014) from BioLegend; APC-labeled CD115 (AFS98, #135509), gp130 (4H1B35, #149405), F4/80 (BM8, #123116), CD27 (LG.3A10, #124211), and PD-L2 (TY25, #107206) from BioLegend; Alexa 700-labeled Sca1 (D7, #108141) from BioLegend; APC/Cy7-labeled Ly6G (1A8, #127624), Ly6C (1G7.G10, #128025), I-A/I-E (M5/114.15.2, #107627), and B220 (RA3-6B2, #103224) from BioLegend; APCVio770-labeled Ly6d (49-H4, #130-115-315) from Miltenyi Biotec (Germany); BV421-labeled CD115 (AFS98, #135513), F4/80 (BM8, #123131), and c-Kit (2B8, #105827) from BioLegend; BV605-labeled CD19 (1D3, #115539) from BioLegend; BV650-labeled CD11b (M1/70, #101239) from BioLegend; PerCP/Cy5.5-labeled Streptavidin (#405214) from BioLegend; and PE-labeled CCR2, either Y15-488 from BD Biosciences (San Jose, CA, USA, #475301) or clone 475301 from R&D Systems (Minneapolis, MN, USA,FAB5538P). Dead cells were excluded using propidium iodide staining. Data were acquired on a CytoFLEX S flow cytometer (Beckman Coulter, Brea, CA, USA) with standardized voltage settings and analyzed using FlowJo software (BD Biosciences).

### Intracellular staining for phosphorylated proteins

For phospho-STAT3 analysis, 1 × 10⁵ cCLPs were stimulated with 100 ng/mL IL-6 for defined time intervals (0–60 min) at 37 °C. Reactions were terminated by the addition of ice-cold MACS buffer. Cells were fixed using CytoFix™ fixation buffer (BD Biosciences), permeabilized with cold methanol, and stained with Alexa 647-conjugated anti-phospho-STAT3 (Ser727) antibody (BioLegend, 698913, clone#A16089B). Finally, cells were washed with FACS buffer and analyzed using a BD FACSCanto™ flow cytometer (BD Biosciences).

### May-Grünwald Giemsa staining

Cell morphology was assessed using May-Grünwald-Giemsa staining of cytocentrifuge preparations. Briefly, sorted populations were suspended at 1 × 10⁵ cells/mL and applied to glass slides using a Cytospin 3 centrifuge (200 rpm, 2 min). After methanol fixation, slides were sequentially stained with May-Grünwald solution (Merck, Darmstadt, Germany, 1014240100) for 3 min and diluted Giemsa solution (Merck, 1092040100) for 15 min. Images were subsequently acquired with a Keyence BZ-X800 microscope (Osaka, Japan).

### Stimulation with toll-like receptor (TLR) ligands

BMDMs were harvested on day 6 using ice-cold PBS/5 mM EDTA, verified for CD11b and F4/80 expression, and re-plated at 2 × 10⁴ cells/well in complete RPMI medium. cCLPs, cCLP-Ms, and BMmono were isolated using FACSAria™ II or FACSAria™ Fusion (BD Biosciences) and plated at 2 × 10⁴ cells/well in 96-well plates in complete RPMI medium. Cells were stimulated with LPS (1 µg/mL), R848 (1 µM), or ODN2395 (1 µM). Culture supernatants were collected after 24 h for cytokine analysis. Cells were harvested simultaneously for flow cytometric assessment of activation markers.

### ELISA

Cytokine levels in culture supernatants were measured using standardized ELISA protocols. Mouse TNF-α, IL-6, and IL-10 were quantified using ELISA MAX™ Standard Sets (BioLegend) according to the manufacturer’s specifications. Wells were reacted with ELISA POD Substrate TMB Kit (Nacalai Tesque, 05298-80), and the reaction was stopped using 2 N H_2_SO_4_. Absorbance was measured at 450 nm using an iMark microplate reader (Bio-Rad Laboratories, Hercules, CA, USA).

### Efferocytosis assay

Apoptotic target cells were generated from freshly isolated mouse thymocytes (4 × 10⁷) treated with 2 μM dexamethasone (Sigma, D1159) for 18 h. Apoptotic cells were labeled with 1 μM carboxyfluorescein succinimidyl ester (CFSE; BioLegend, 423801), washed extensively, and resuspended at 2 × 10⁷ cells/mL. cCLPs, cCLP-Ms, or their differentiated derivatives were plated at 2 × 10⁵ cells/mL in 96-well plates and cultured overnight. Where indicated, cells were pre-treated with 1 μM Latrunculin B (Calbiochem, 428020) for 30 min to inhibit actin polymerization. CFSE-labeled apoptotic cells were added at a 10:1 ratio (apoptotic cells: phagocytes) and co-cultured for 3 h at 37 °C. Phagocytosis was quantified by flow cytometry, measuring the percentage of CD11b⁺ cells that had acquired CFSE fluorescence.

### M1/M2 polarization

cCLP-Ms were harvested, washed thoroughly, and cultured for 3 days at 2 × 10^5^/mL with rm M-CSF (10 ng/mL), LPS (100 ng/mL) + IFN-γ (10 ng/mL), or IL-4 (10 ng/mL). Polarization was assessed by flow cytometric analysis of characteristic markers including PD-L1 (M1) and PD-L2 (M2).

### RNA-sequencing analysis

Bone marrow cells from C57BL/6 mice (7–18 weeks old) were prepared to isolate CD11b⁺Ly6C⁻Ly6G⁺ neutrophils (BMneu) and CD11b⁺CD115⁺CCR2⁺Ly6C^hi^Ly6G⁻ monocytes (BMmono). Additionally, bone marrow cells were cultured with 20 ng/mL M-CSF for 4 days to generate CD11b⁺ cultured macrophages (BMDMs). cCLPs (passages 6–21, cultured in vitro for 1–2.5 months) and those stimulated with 10 ng/mL FL/IL-6/SCF for 1 day (D1) were collected for RNA extraction. Similarly, cCLPs cultured with 10 ng/mL FL/IL-6/SCF for 3 days (D3) were harvested to isolate CD11b⁺CD115⁺CCR2⁺Ly6C⁺Ly6G⁻ cells. Cell sorting was performed using FACSAria™ II or FACSAria™ Fusion (BD Biosciences).

Total RNA was extracted using the RNeasy Micro Kit (QIAGEN, Germany, 74004) according to the manufacturer’s instructions. Three biological replicates per group were subjected to RNA sequencing using the BGISEQ platform with a PE100 sequencing length (BGI). Library preparation and sequencing were performed by BGI using a DNBSEQ sequencer. Raw sequencing reads underwent quality control and adapter trimming using fastp (v0.21.0). Trimmed reads were mapped and quantified using RSEM (v1.3.1), with STAR (v2.7.10) as the aligner. Expression data were imported into iDEP.96 [46], normalized using CPM (counts per million), and log-transformed with EdgeR [log₂(CPM + 4)] for principal component analysis (PCA) and k-means clustering analysis.

Differentially expressed genes (DEGs) were identified using DESeq2 (v1.34.0), applying thresholds of |log₂ fold-change (FC) | > 1.0 and adjusted *p*value < 0.05. These DEGs were visualized using heatmaps and scatter plots. For functional enrichment analysis, Metascape (http://metascape.org) [47] was used to perform Gene Ontology (GO) annotation analysis and tissue/cell type association analysis based on the PaGenbase database [48].

### CRISPR/Cas9-mediated gene editing

Synthesized Alt-R® CRISPR-Cas9 crRNAs, Alt-R® CRISPR-Cas9 tracrRNA (#1072534), Alt-R® S.p. Cas9 Nuclease V3 (#1081059), and the electroporation enhancer (#1075916) were purchased from Integrated DNA Technologies (IDT; Coralville, IA, USA) and assembled into RNA ribonucleoprotein (RNP) complexes according to the manufacturer’s protocol. The crRNAs for *Cebpa* (5’-TAGAAGTCGGCCGACTCCAT-3’), *Cebpb* (5’-GAGGCTCACGTAACCGTAGT-3’), and *Id1* (5’-GCATGTAATCGACTACATCA-3’) were validated by IDT. cCLPs (1 × 10⁵) were suspended in 80 µL of Opti-MEM, mixed with Cas9-RNP complexes, and electroporated using the NEPA21 system (NEPAGENE, Chiba, Japan) at 275 V and 2.5 ms pulse. Following electroporation, cells were cultured in 24-well plates containing OP9 cells in cCLP culture medium for 4 days to allow recovery. The cells were harvested and re-plated at 1 cell/well in a 96-well flat-bottom plate coated with OP9 in cCLP culture medium for clonal expansion over seven days. The resulting single-cell clones were validated through T7 endonuclease I assay (New England Biolabs, Ipswich, MA, USA) and Sanger sequencing of the targeted regions, followed by functional assessment of differentiation capacity.

### Retroviral vector construction and transduction

Total RNAs were extracted from cells using TRI reagent (Molecular Research Center, Cincinnati, OH, USA, TR118), and cDNAs were synthesized using ReverTra Ace (Toyobo, Osaka, Japan, TRT-101) with oligo-dT_18_ (Eurofins) and random primers (Invitrogen, 48190011). The long isoform of mouse *Cebpb* (LAP, transcript variant 1, NM_001287738.1) was amplified from cCLP-M-derived cDNA using KOD-Plus-NEO polymerase (Toyobo, KOD-401) with specific primers (forward: 5ʹ-aggcgccggaattcgttaacctcgagatggaagtggccaacttctactac-3ʹ; reverse: 5ʹ-gaggggcggaattgatcccgctcgagctagcagtggcccgccgag-3ʹ). The amplified PCR product was inserted at the *Xho*I site of the pMSCV-IRES-GFP retroviral vector (pMIG) using NEBuilder HiFi DNA Assembly Master Mix (New England Biolabs, E2621S). The construct was verified by Sanger sequencing and transfected into Plat-E cells using PEI-MAX (Polyscience, Warrington, PA, USA, 24765-100). Viral supernatants were harvested 72 h post-transfection. For retroviral transduction, 1.2 × 10⁵ cCLPs were suspended in a mixture containing equal volumes (500 μL each) of viral supernatant and cCLP medium. Spin infection was performed at 1150 × *g* and 30 °C for 3 h. The infected cells were washed twice, and 1 × 10⁴ cells were cultured in 200 μL complete RPMI medium in 96-well plates pre-coated with OP9 for 4 days. Half of the cell suspension (100 μL) was collected for flow cytometric analysis.

### Induction of IgE-induced chronic allergic inflammation (IgE-CAI)

IgE-CAI was induced as previously described [49] by intravenously administering 300 μg of anti-TNP-IgE to wild-type mice, followed by intradermal injection of 10 μg TNP-conjugated ovalbumin (TNP-OVA) into the right ear as an allergen. As a control, 10 μg of unconjugated ovalbumin (OVA) was administered to the left ear. The value of the differences in ear thickness (right—left) was calculated for the evaluation of inflammation. For cell transfer experiments, 1 × 10⁶ cells were intradermally co-injected into the ear along with Flt3L (10 ng) simultaneously with the allergen. cCLPs and cCLP-Ms were isolated via IMag system with biotinylated anti-CD45 and anti-CD115, respectively, followed by BD IMag™ Streptavidin Particles Plus (BD Biosciences) before labeling with or without cell trace violet (CTV, Thermo Scicentific, C34557) according to the manufacturer’s protocol.

### Statistical analysis

Cells and mice were randomly assigned to each treatment group and experiment. No statistical method was used to predetermine sample size. Sample size was chosen empirically based on our previous experience in the calculation of experimental variability. Data were analyzed with the GraphPad Prism software version 7.0. *p*values were calculated by one-way or two-way ANOVA. Statistical significance was determined with alpha <0.05 and presented as *, <0.05; **, <0.01; ***, <0.001; ****, <0.0001.

## Supplementary information

Supplementary information

Supplementary Table 1

Supplementary Table 2

## Data Availability

The datasets generated during and/or analysed during the current study are available in the NCBI Sequence Read Archive (SRA) repository, https://www.ncbi.nlm.nih.gov/bioproject/PRJNA1225313.
