# Supplementary material for: IL-6-C/EBPβ signaling drives monocytic differentiation of murine cultured lymphoid progenitors with immunoregulatory properties
Source: Cell Death Dis. 2025 Aug 12;16(1):612. doi: 10.1038/s41419-025-07930-4 (PMC12343812; doi:10.1038/s41419-025-07930-4)
Supplement: Supplementary file 1 — Supplementary information [file 41419_2025_7930_MOESM1_ESM.docx]

**Supplementary figures**

**L-6-C/EBPβ signaling drives monocytic differentiation of murine cultured lymphoid progenitors with immunoregulatory properties**

**Yohei Kawano¹*, Nozomi Katsuya¹, Mizuki Moriyama¹, Shun Ohki¹, Yasuo Kitajima¹, and Tomoharu Yasuda¹**

¹ Department of Immunology, Graduate School of Biomedical and Health Sciences, Hiroshima University, 1-2-3 Kasumi, Minami-Ku, Hiroshima 734-8551, Japan.

**
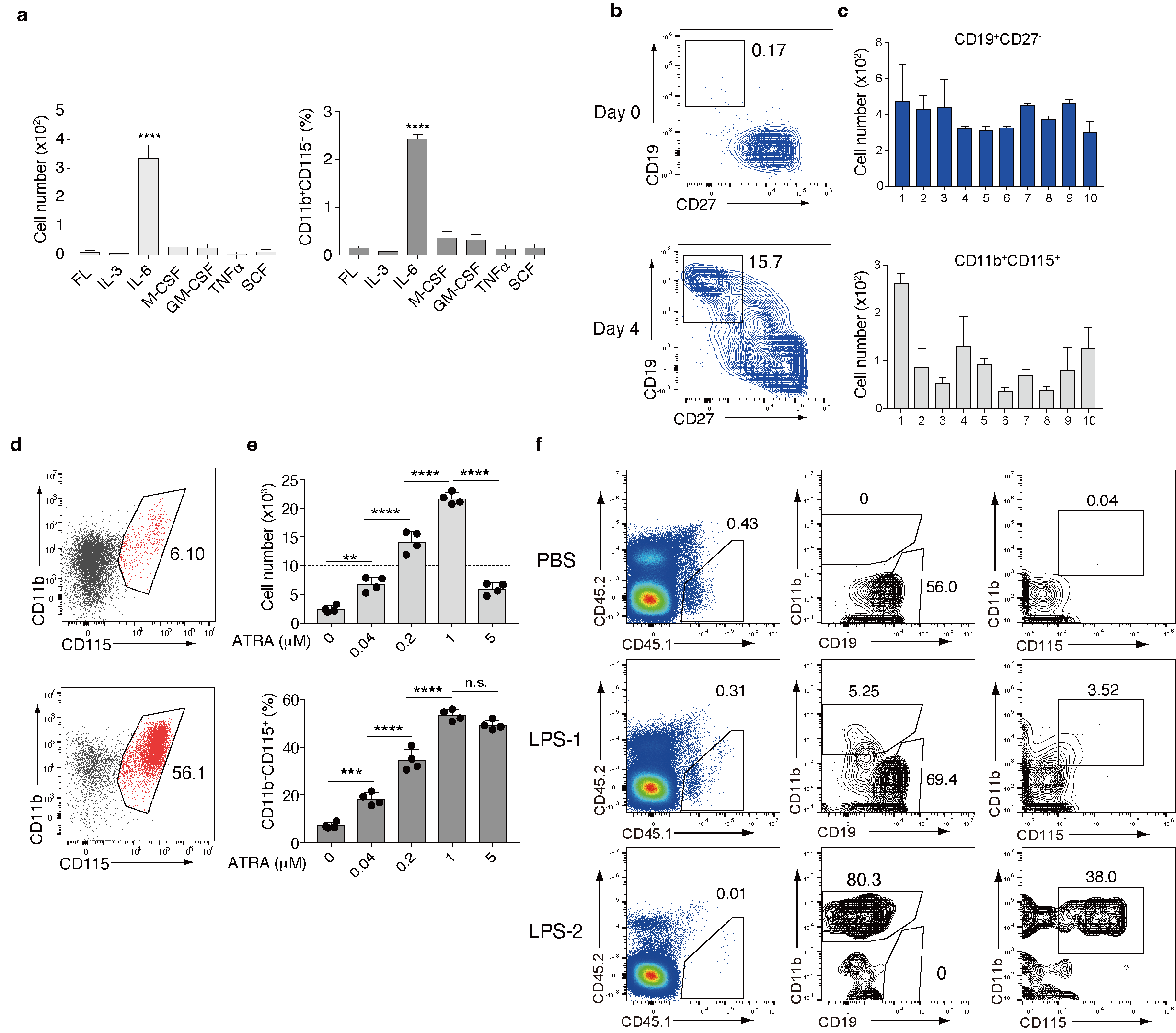
**

**Supplementary Figure 1.** Generation of CD11b^+^CD115^+^ myeloid lineage from cCLPs from cryopreserved stocks and maintained for two months

(**a**) Cell number (*upper panel*) and percentage (*lower panel*) of CD11b^+^CD115^+^ cells on day 3 after culture of 3,000 cCLPs with the indicated cytokines in the presence of FL (n=3, each). (**b**) Representative FACS profiles showing CD19 and CD27 expression on day 0 (*upper panel*) and day 4 (*lower panel*) after culture of 10,000 cCLPs with FL (2.5 ng/mL) and IL-7 (10 ng/mL) (**c**) Numbers of CD19^+^CD27^-^ B cells (*upper panel*) and CD11b^+^CD115^+^ myeloid cells (*lower panel*) differentiated under B cell or myeloid culture conditions, respectively, using 10 different types of FBS (n=2, each). (**d**) Representative FACS profiles on CD11b and CD115 expression day 3 after culture of 10,000 cCLPs with FL/IL-6/SCF in the presence (*lower panel*) or absence (*upper panel*) of ATRA (1 μM). The percentage in the gate is shown. (**e**) Cell number (*upper panel)* and percentage (*lower panel*) of CD11b^+^CD115^+^ cells at the different concentration of ATRA are shown (n=4, each). Dashed line indicates the initial input number of cCLPs. (**f**) CD45.1⁺ cCLPs (2x10^6^) were transferred into sublethally irradiated (6 Gy) CD45.2⁺ recipient mice, followed by intraperitoneal injection of either PBS (*upper panels*) or 5 mg/kg of LPS (*middle and lower panels*). On day 7, bone marrow cells were analyzed for the presence of donor-derived cells. Data are mean ± SD with statistical significance determined by one-way ANOVA with multiple comparisons. The *p*-values are represented as **, <0.01; ***, <0.001; ****, <0.0001. n.s., not significant.


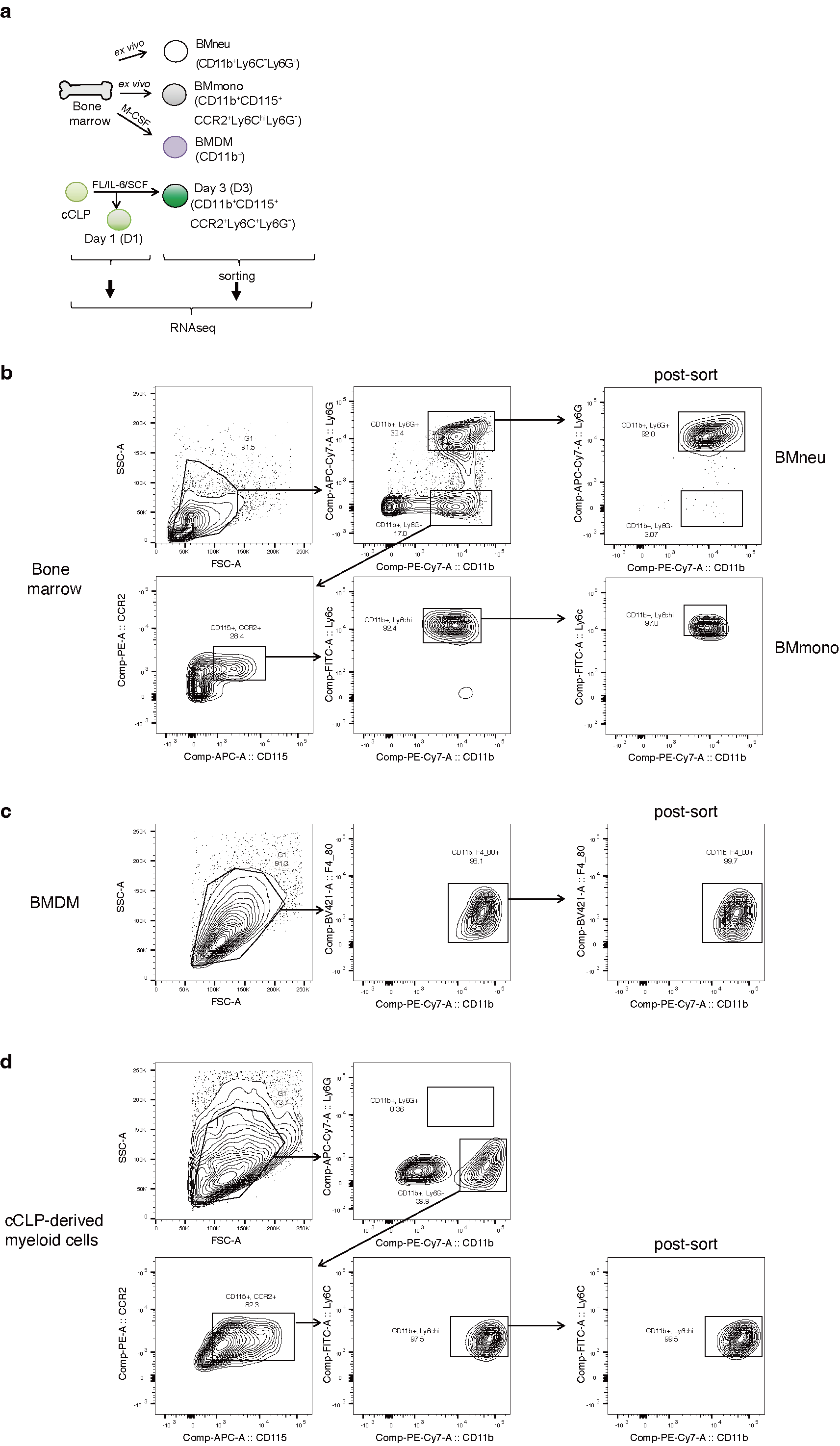


**Supplementary Figure 2.** Isolation of cCLP-derived myeloid cells, BMDM, CD11b^+^Ly6G^+^ neutrophils (BMneu) and CD11b^+^Ly6C^hi^ monocytes (BMmono) from a mouse bone marrow for RNA-sequencing analysis

(**a**) Schematic representation on sample preparation for RNA-sequencing analysis (n=3, each). (**b-d**) Representative FACS plots for the isolation of the indicated cell populations. Right panels show the FACS plots on the indicated marker expression for post-sort samples.


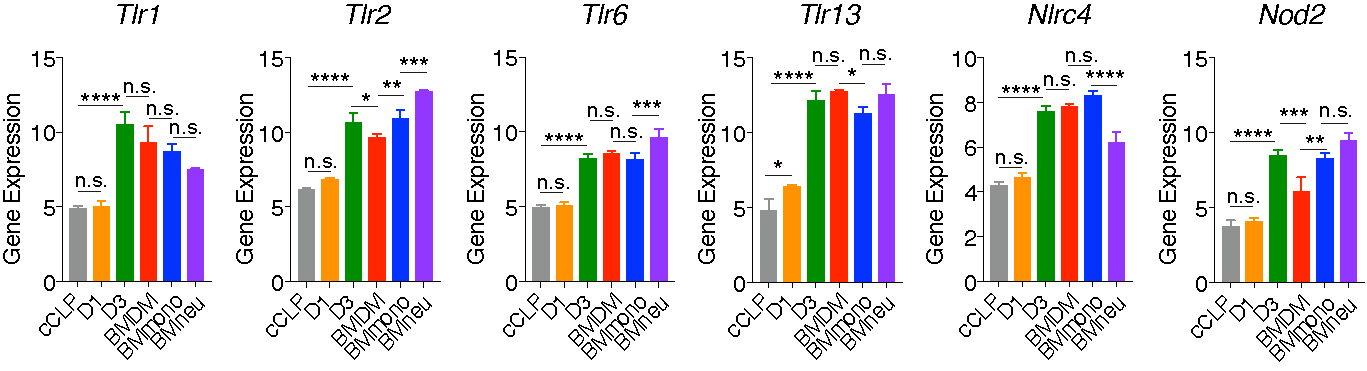


**Supplementary Figure 3.** Representative gene expression related to “innate immune response” in Cluster G

The expression levels of the indicated genes derived from RNAseq data are shown as log_2_ (CPM+4) on y-axis. Data are mean ± SD with statistical significance determined by one-way ANOVA with multiple comparisons. The *p*-values are represented as *, <0.05; **, <0.01; ***, <0.001; ****, <0.0001. n.s., not significant.

**
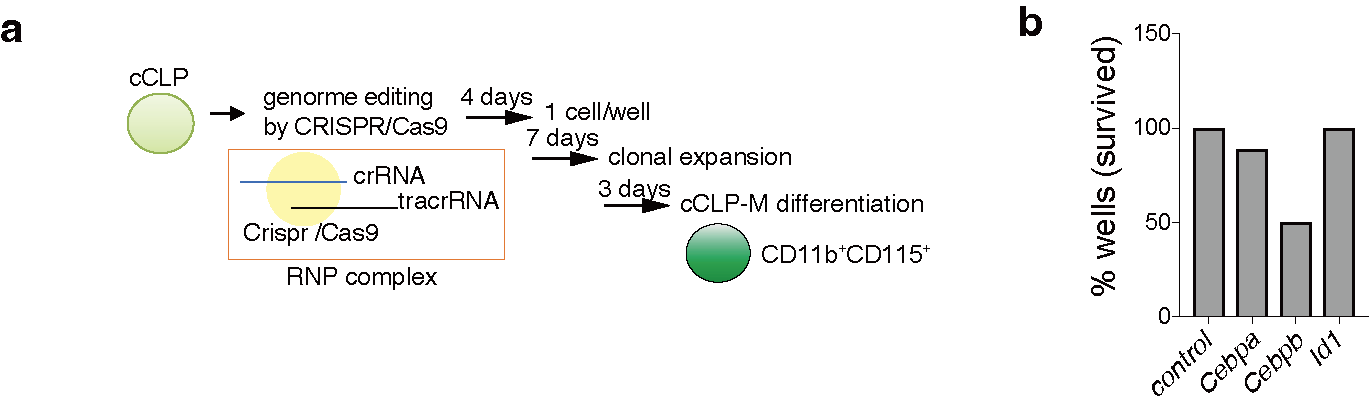
**

**Supplementary Figure 4.** CRISPR/Cas9-mediated gene editing in cCLPs for their differentiation into cCLP-M

(**a**) CRISPR/Cas9-mediated gene editing in cCLPs (**b**) Frequency of wells with more than 100 live cells after 7 days-expansion of single clones of cCLPs where indicated genes were targeted by Crispr/Cas9.


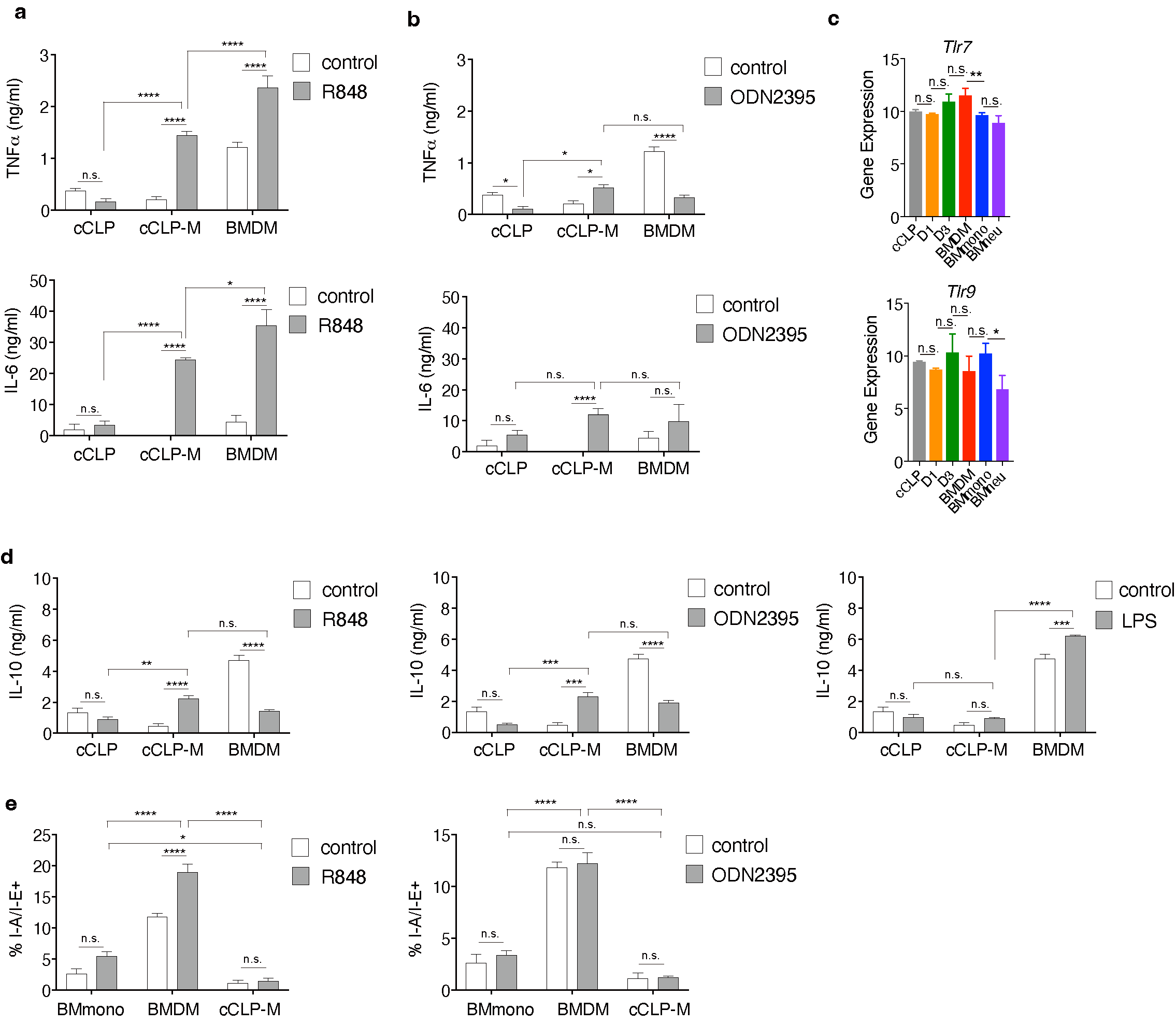


**Supplementary Figure 5.** *In vitro* responses of cCLP-Ms to different TLR ligands

The indicated cells were stimulated with LPS, R848, ODN2395, or medium alone (control) for 24 hours. Culture supernatants and cells were harvested for ELISA and FACS, respectively. (**a, b**) TNFα (*upper panels*) and L-6 production (*lower panels*) after stimulation with (*filled bars*) or without (*open bars*) R848 or ODN2395. Error bars indicate mean± SD from three-independent experimental results. (**c**) Gene expression for *Tlr7* and *Tlr9* in the indicated cells before stimulation (n=3, each). The expression levels derived from RNAs-eq data are shown as log_2_ (CPM+4) on y-axis. (**d**) IL-10 production after stimulation with (*filled bars*) or without (*open bars*) LPS (*left panel*) R848 (*middle panel*) or ODN2395 (*right panel*) (n=3, each). (**e**) The percentage of I-A/I-E^+^ cells after stimulation with (*filled bars*) or without (*open bars*) R848 (*left panel*) or ODN2395 (*right panel*) (n=3, each). Data are mean ± SD with statistical significance determined by one-way ANOVA (in **c**) or two-way ANOVA (in **a**, **b**, **d**, **e**) with multiple comparisons. The *p*-values are represented as *, <0.05; **, <0.01; ***, <0.001; ****, <0.0001. n.s., not significant.


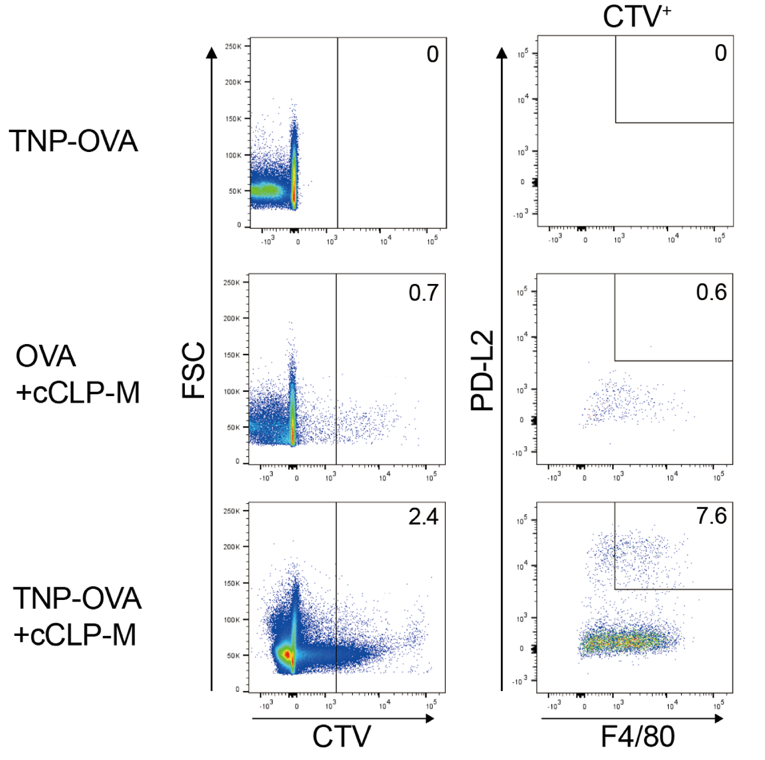


**Supplementary Figure 6.** Transferred cCLP-Ms differentiate into M2 macrophages in skin lesion

Representative FACS plots showing F4/80^+^PD-L2^+^ cells (*right panels*) within cell trace violet (CTV)^+^ gate (*left panels*) in the ear skin two days after the antigen challenge with TNP-OVA alone (*upper panels*), or with OVA (*middle panels*) or TNP-OVA (*lower panels*) in combination with the transfer of CTV-labeled cCLP-M into wild-type mice.
